# Efficacy of Cognitive Behavioral Therapy With Local Wisdom and Web-Based Counseling on Generalized Anxiety Disorders and Functional Gastrointestinal Disorders in Adolescent College Girls: Protocol for a Randomized Controlled Trial

**DOI:** 10.2196/50316

**Published:** 2023-08-22

**Authors:** Zadrian Ardi, Chiedu Eseadi, Elsa Yuniarti, Frischa Meivilona Yendi, Arina Widya Murni

**Affiliations:** 1 Department of Guidance and Counselling Faculty of Education Universitas Negeri Padang Indonesia; 2 Department of Educational Psychology Faculty of Education University of Johannesburg Johannesburg South Africa; 3 Biology Department Faculty of Mathematics and Natural Sciences Universitas Negeri Padang Indonesia; 4 Internal Medicine Department Subdivision of Psychosomatic Medicine Universitas Andalas Padang Indonesia

**Keywords:** adolescent college girls, cognitive behavioral therapy, CBT, functional gastrointestinal disorders, FGIDs, generalized anxiety disorders, GADs, local wisdom, web-based counseling

## Abstract

**Background:**

The high academic demands experienced by students will often have an impact on the quality of their mental and physical health. The most common health problems reported are gastrointestinal disorders. This condition tends to manifest in the emergence of generalized anxiety disorders (GADs) and reduces the quality of life and productivity. The population that experiences this disorder the most is female adolescents, and this condition occurs in both South African and Indonesian populations. The use of drugs, especially benzodiazepines, often causes psychological conditions as side effects. For this reason, it is necessary to have a solution in the form of a targeted and efficient approach to reduce psychological symptoms that arise from functional gastrointestinal disorders (FGIDs) in the form of anxiety.

**Objective:**

The purpose of this study is to produce and implement a counseling intervention model to assist female students with GADs caused by FGID factors using an approach combining cognitive behavioral therapy (CBT), web-based counseling, and local wisdom in Indonesian and South African populations.

**Methods:**

The research subjects will comprise 118 female adolescent students from Indonesia and 118 female adolescent students from South Africa, making a total sample of 236 participants, and the study will use a prospective, parallel randomized controlled trial design. The recruitment process will begin in July 2023, and the trial will begin in August 2023. The posttest assessment data gathering will take place by November 2023. Questionnaires that will be used in this study include the Functional Gastrointestinal Disorder Checklist (FGI-Checklist) to collect data related to FGIDs and the Generalized Anxiety Disorder 7-item (GAD-7) to measure the anxiety conditions experienced by respondents.

**Results:**

By adopting the intention-to-treat principle, there will be significant mean changes in GAD scores and FGID scores after exposure to this combined approach in the Indonesian and South African populations. Implementing this comprehensive intervention will improve the students’ psychological symptoms related to FGIDs and ultimately enhance their overall well-being.

**Conclusions:**

This study will develop and implement a model of counseling intervention for female students with GADs obtained from FGIDs using a combination approach to CBT, web-based counseling, and local wisdom in both the Indonesian and South African populations. The trial findings will contribute to our understanding of the effects of CBT combined with local wisdom and web-based counseling approaches that mental health counselors can use to treat GAD-affected adolescent girls who have FGIDs.

**Trial Registration:**

UMIN Clinical Trial Registry UMIN000051386; https://tinyurl.com/yjwz8kht

**International Registered Report Identifier (IRRID):**

PRR1-10.2196/50316

## Introduction

### Background

Students experience various changes and challenges over the period of their studies. The high academic demands that students must meet and the tendency for weak control over health impact students’ conditions [1,2]. Various mental health disorders also tend to increase during an individual’s time as a student [3-5]. This condition tends to cause health problems and affects students’ quality of life and mental condition. Disorders that appear commonly in students are functional gastrointestinal disorders (FGIDs) [6,7], which are characterized by abdominal pain, dysphagia, dyspepsia, diarrhea, constipation, bloating, and acute gastrointestinal (GI) disturbances [7-9]. Based on data from the Ministry of Health of the Republic of Indonesia in 2020, FGIDs are included in the list of 10 diseases that affect adolescents the most in Indonesia, with a prevalence of up to 40.85% [10]. FGIDs appear in conditions without an organic basis and are more likely to occur due to impaired communication between the brain and the gut. In other words, disturbances in digestive function are reciprocal. Individuals with FGIDs tend to experience anxiety disorders, while individuals with dominant anxiety conditions will experience digestive disorders [8,11-14]. Treating FGIDs based on the latest findings generally still relies on drug therapy, but this therapy tends to produce other side effects subsequently experienced by the patient [15]. In addition, FGID treatment, which essentially does not produce organic conditions, is time-consuming and expensive. In addition, the patient’s anxiety condition often does not subside and worsens [13,16].

When viewed from a demographic perspective, anxiety disorders as a result of FGIDs are often experienced by the female population during their reproductive years [17,18]. It has been found in population-based case-control studies that women with FGIDs experience a more negative impact on their daily functioning than their male counterparts and other women without this condition [12]. In the South African population, 8% of the total population experienced this; in the KwaZulu-Natal province of South Africa, it was found that among 201 rural patients who presented with one type of FGID (functional dyspepsia), most patients (74.13%) [19] reported anxiety disorders. The impact is that if anxiety conditions and FGIDs are not handled properly, this will decrease students’ productivity and quality of life. Generally, this impact will result in a decrease in students’ opportunities for achievement and learning outcomes to the point of being unable to continue their studies [9,13,16,20]. Anxiety resulting from FGIDs also contributes significantly to the decline in quality of life. It impacts both the student’s college life in terms of their learning, social, and personal lives, and the condition of their future career. Various attempts to alleviate this problem have been made but have not yielded significant results. Among others, a pharmacotherapeutic approach relieves anxiety symptoms that arise [15,21]. However, the use of drugs, especially benzodiazepines, often causes side effects on psychological conditions. For this reason, it is necessary to have a solution in the form of a targeted and efficient approach to reduce psychological symptoms that arise from FGIDs in the form of anxiety [22-41]. With a specific target on anxiety sufferers in the female population of student age, it is deemed appropriate to provide a cognitive behavioral therapy (CBT) approach combined with the implementation of web-based counseling and local wisdom. The advantage of this approach is that the assessment process can be carried out in a remote setting using web-based counseling technology developed in the author’s previous research, then combined with an approach that has proven effective in conducting behavioral change therapy and emphasizes the implementation of local wisdom. In addition, the combination of these 3 elements will be able to spur positive changes in the anxiety experienced by female students with FGID conditions.

### Objective

The purpose of this study is to produce and implement a counseling intervention model to assist female students with generalized anxiety disorders (GADs) caused by FGID factors using an approach combining CBT, web-based counseling, and local wisdom in Indonesian and South African populations.

### Hypothesis

Based on the understanding that students face various challenges and changes during their university studies, including the high academic demands and potential impact on their health, particularly in terms of mental health, it is important to address these issues effectively. One common disorder among students is FGIDs, which not only affect their physical well-being but also have an impact on their quality of life and mental state. Additionally, anxiety disorders often coexist with FGIDs, further exacerbating the negative effects on students’ overall well-being. Considering the prevalence of FGIDs and anxiety disorders among female students, it is crucial to develop an intervention model that combines CBT, web-based counseling, and local wisdom. Thus, this study hypothesizes that there will be significant mean changes in GAD scores and FGID scores after exposure to this combined approach in the Indonesian and South African populations. Implementing this comprehensive intervention is expected to improve the students’ psychological symptoms related to FGIDs and ultimately enhance their overall well-being.

## Methods

### Research Design

This study is developmental research involving the systematic and conceptual design of a psychological treatment approach. The development model that will be used as the basis for this research activity is one adapted from the ADDIE (Analysis, Design, Development, Implementation, and Evaluation) model [42,43]. A prospective, parallel randomized controlled trial design involving pre- and posttesting of a treatment group and a control group will be used by the researchers [44]. At the end of a diagnostic interview conducted by the researchers, participants who are considered eligible for the study will be randomly assigned to 1 of 2 groups: the web-based CBT group or the usual care control group.

### Research Subjects

The research subjects will consist of female adolescent students in Indonesia and South Africa (aged 15-17 years); 118 people from each country will be recruited from general practice or family medicine clinics as well as high schools within each study area to arrive at an overall sample of 236 participants for this study. An initial assessment will be conducted on 1000 potential participants (500 adolescent girls per country), who will be screened according to the research criteria so that a representative sample for the intervention may be obtained. The results of a recent study [45] found that 44.1% of participants were able to complete a CBT program using a web-based approach and that the average completion rate for the 12 sessions was 7.8 (65%). Therefore, we will take into account the attrition rate of 55.9% by including 55.9% extra participants in our initial sample for each group produced by the G*power (Heinrich-Heine-Universität Düsseldorf) sampling program [46]. To account for the potential effects of missed sessions in a standard web-based CBT program, as shown by recent research [45], 4.2 sessions (35%) will be added to the 12-session program, which will result in approximately 16 sessions (to be delivered over 4 months). Based on the G*power calculation, the required sample size is 38 participants per group (85% statistical power; medium effect size Cohen *d*=0.5; a priori probability level of 0.5) for a linear mixed model (LMM) analysis. Given the presumed attrition rate, we will recruit 59 participants per group in each country.

### Randomization and Masking

This study will use a computer-generated random list obtained from Random Allocation Software [47] to randomize groups using a simple randomization technique by 2 biostatisticians (1 for each country) who are not part of the research team to ensure assignment concealment. Each country’s research team is expected to have 59 research subjects in the treatment group and 59 research subjects in the control group, totaling 118 adolescent girl participants from each country. The group randomization process will be concealed from the study subjects. Thereafter, the researchers will notify subjects of their group membership through invitations to participate in the treatment intervention or usual care sessions.

### Treatment Activities and Procedures

This research will be reported in line with the guidelines for the Consolidated Standards of Reporting Trials (CONSORT) [48]. The study was registered in a clinical trials registry before the recruitment of the first participant. A 16-session CBT manual incorporating web-based counseling techniques and principles and local wisdom approaches to the treatment of GAD among adolescent college girls with FGID symptoms will be developed by the research team for this study. The research team will adapt evidence-based techniques and procedures from existing CBT intervention manuals for GAD and FGID treatments with a focus on cultural components, traditions and values, and local wisdom, as well as web-based counseling techniques and processes, to address GAD and FGID symptoms among adolescent college girls. Each web-based session for the treatment group as well as the usual care session for the control group in each country (study site) is expected to last for 90 minutes each, once a week for 4 months. To assess the efficacy of CBT combined with local wisdom and web-based counseling approaches, we used the usual care as a comparison group. This study defines usual care as whichever treatment for GAD and FGIDs adolescents receive from their regular health care providers or therapists. This form of comparison group was selected to appraise the effects of CBT combined with local wisdom and web-based counseling approaches against the treatments that would normally be available without this intervention. The use of usual care as a comparison group also contributes to the viability of the trial since other clinicians are not required to be trained in a particular approach to comparator therapy. Due to this study’s design, it is possible to compare the effects of CBT combined with local wisdom and web-based counseling methods with the usual practice in GAD treatment for adolescent girls with FGIDs. Three specialist raters in each country will evaluate the treatment’s integrity. During the project, 2 postgraduate-level psychologists with a master’s degree will serve as research assistants in each country. Two cognitive behavioral therapists with a doctorate in counseling psychology will provide the web-based CBT intervention for the treatment group, while the usual care intervention for the control group in each country will be provided by 2 CBT practitioners with doctorate degrees in psychology. The recruitment process will begin in July 2023, and the trial will begin in August 2023. The posttest assessment data gathering will be undertaken by November 2023. A summary of the treatment intervention is shown in Table 1.

**Table 1:** Summary of the intervention components.

Sessions	Integrated web-based counseling with local wisdom CBT^a^
Initial assessment (session 1)	In the first session, the counselor and clients will engage in an initial assessment to identify the presenting issues and set goals for therapy. This assessment typically takes 1 session. This stage involve the use of a web-based assessment system.
Sessions 2-3	Psychoeducation (2 sessions): Over 2 sessions, the counselor provides educational materials and resources to the clients through text, videos, or web-based sources. The counselor explains the concepts of CBT, the connection between thoughts, feelings, and behaviors, and introduces the techniques and strategies that will be used in therapy.
Sessions 4-5	Identification of negative thought patterns (2 sessions): This process involves self-observation, reflection, and discussion, taking into account the perspective of cultural values and local wisdom. By incorporating cultural perspectives and values, the counselor aims to create a culturally sensitive and inclusive environment that respects the clients’ cultural background. The exploration and identification of these patterns typically take 2 sessions, allowing sufficient time for the client to reflect and discuss their experiences within the context of their cultural beliefs and values.
Sessions 6-9	Restructuring negative thoughts (4 sessions): Once negative thought patterns are identified, the counselor and clients collaborate to restructure these thoughts and replace them with more realistic, balanced, and positive thoughts, integrating the principle of “nature as the master teacher” from Minangkabau culture in Indonesia. This process involves techniques such as realistic thinking, thought replacement, and asking challenging questions while drawing inspiration from nature’s wisdom. By incorporating local wisdom, the counselor helps the clients develop a deeper understanding and connection with their cultural roots. In this respect, the Ubuntu counseling model as a local wisdom approach will be integrated to assist the South African adolescents during the treatment sessions since it involves integrating African culture into the therapeutic process in that it recognizes the relevance of culture to patient mental health and self-identity. Several group processes are emphasized in the Ubuntu counseling model, including communality, cooperation, and oneness among others. Several traditions and values are also intrinsic to the Ubuntu counseling model, and they determine how mental disorders are understood and treated in South Africa.
Sessions 10-13	Development of coping skills (4 sessions): The counselor assists the clients in developing healthier coping skills. This includes stress management strategies, relaxation techniques, problem-solving skills, and fostering adaptive thinking patterns. It typically takes 4 sessions to introduce and practice these skills.
Sessions 14-15	Assignments and exercises (2 sessions): Throughout therapy, the counselor assigns tasks and exercises for the clients to apply the concepts and skills learned in sessions to their daily lives. The counselor may review progress and provide feedback in 2 dedicated sessions for discussing and reviewing assignments.
Session 16	Monitoring and feedback (1 session): The counselor uses web-based monitoring tools, such as questionnaires or web-based daily journals, to track changes in the clients’ thoughts, feelings, and behaviors. In 1 session, the counselor provides constructive feedback based on the information gathered and the progress made.

^a^CBT: cognitive behavioral therapy.

### Inclusion and Exclusion Criteria

To be considered eligible, participants must (1) be between the ages of 15 and 17 years, (2) be experiencing moderate to severe GAD and FGID symptoms at baseline, (3) speak and understand English to a reasonable level, (4) be willing to take part in the web-based CBT program or attend the usual care program, (5) have been taking continuous doses of anxiety medications for at least two months before the commencement of the program, (6) have a smartphone or tablet device that is capable of running Whatsapp, Zoom, or the Konselo counseling app and have access to the internet, which is necessary to participate in the program sessions, and (7) demonstrate the ability to commit to the expectations of the trial. Some conditions will exclude participants from the study. This includes (1) participants who cannot answer questions in the assessment tools on their own with a reasonable degree of English, (2) participants affected by psychiatric or medical comorbidities that would severely impact their ability to participate or adhere to the program schedule, (3) persons who are currently receiving CBT treatment, (4) individuals who have practiced meditation regularly or previously, (5) a lack of internet access or the inability to use a device that is capable of connecting to the internet, and (6) inability to commit to the expectations of the trial. In each country, 2 postgraduate-level research assistants with a master’s degree in psychology will be trained to screen prospective participants using these inclusion and exclusion criteria by telephone. On completion of the screening process and after it has been determined that participants are eligible for the study, they will be invited to complete a diagnostic interview with the research investigators in each study area to additionally validate their eligibility for the study. Participants will be reached by phone in line with the schedule of the program as soon as it has been determined that they are eligible to participate in the research study.

### Outcome Measures

Questionnaires that will be used in this study include the Functional Gastrointestinal Disorder Checklist (FGI-Checklist) [49] to collect data related to FGIDs and the Generalized Anxiety Disorder 7-item (GAD-7) screening tool [50] to measure the anxiety conditions experienced by respondents.

The FGI-Checklist includes the 20 most common FGID symptoms, which encompass a series of symptoms across the GI tract [49]. Five subscales contribute to the total score of the FGI-Checklist, including abdominal and bowel syndrome (7 items; Cronbach α=.86), functional dyspepsia syndrome (5 items; Cronbach α=.87), reflux syndrome (4 items; Cronbach α=.80), esophageal syndrome (3 items; Cronbach α=.69), and nausea and vomiting syndrome (2 items; Cronbach α=.73). Several single items on the FGI-Checklist are used to ascertain the severity of global dyspeptic symptoms, abdominal symptoms, stool frequency, and stool form as per the Bristol stool scale. Thus, it is often seen as a comprehensive symptom assessment scale covering esophageal, epigastric, and abdominal symptoms. It is a self-administered outcome measure with a 7-day recall period [49]. Patients are required to rate the intensity of each symptom using 4 response options to indicate impairment in daily activities due to the symptom as follows: 0=none, 1=mild (not affecting daily activity), 2=moderate (some daily activities are affected), and 3=severe (almost all daily activities are affected). The FGI-Checklist total score, which ranges from 0 to 3, is calculated by averaging the scores of the 20 items. Patients with mild (monthly) to severe (weekly) symptoms can be identified with the FGI-Checklist. A higher score represents more severe symptoms [49].

It has been demonstrated that the GAD-7’s internal consistency is excellent (Cronbach α=.92) and its test-retest reliability is adequate (intraclass correlation=0.83) [50]. The GAD-7 has a 4-point response scale ranging from 0 (not at all) to 3 (nearly every day). Scores between 0 and 4 indicate minimal GAD, 5 to 9 indicate mild GAD, 10 to 14 indicate moderate GAD, and 15 to 21 indicate severe GAD [50]. The GAD-7 also has a strong correlation with patients’ mental health (*r*=0.75), social functioning (*r*=0.46), general perceptions of health (*r*=0.44), bodily pain (*r*=0.36), role functioning (*r*=0.33), and physical functioning (*r*=0.30) [50]. An invaluable feature of the GAD-7 is its ability to assess changes in anxiety symptoms severity over recent weeks (that is, over the previous 2 weeks).

### Data Analyses

The analyses of the data in this study will include descriptive analysis, analysis of the coefficient of agreement between program raters (interrater reliability), and internal consistency analysis using Cronbach α and partial η^2^ (effect size statistics). A sensitivity analysis will also be carried out to avert potential data dredging. The Mauchly sphericity test and Levene equality of variance test will be carried out to ensure that the assumptions of the chosen statistical test are met. LMM will be used to analyze the primary outcome measures statistically based on the principle of intention-to-treat to determine whether considerable changes have occurred over time [51]. By using LMM, participants with incomplete data will be included in the analyses so that treatment effects can be assessed over time (group time interaction) [51]. Using LMM, the treatment group, time, and interactions between the treatment group and time will be considered fixed factors; the covariance structure will be constructed in an unstructured manner. We will ascertain posttest outcomes in terms of mean changes in GAD-7 and FGI-Checklist scores from baseline to 4 months following the pretest. Complier-adjusted causal effect (CACE) analysis will be conducted based on the causal framework and estimation methods to account for variations in treatment effect due to variations in treatment adherence between the groups [51,52]. We will calculate CACE as the difference between the mean GAD-7 and FGI-Checklist scores of compliers in the treatment group as well as the control group. To be considered a complier, a participant must have attended 80% or more of the program sessions, which is a frequently used threshold [51,53]. A bootstrapping technique will be used to determine standard errors. Statistical analyses will be completed using R (R Core Team) to analyze the CACE data and SPSS (version 29; IBM Corp) for the rest of the analyses. *P* values of .05 or less will be considered statistically significant.

### Ethics Approval

Each participant will be informed about their freedom to withdraw from the research project at any point in time without consequences. The research team will obtain web-based informed consent from the participating female adolescents, and their right to privacy will be respected. Parental consent will be sought. The project will be conducted in line with relevant ethical guidelines of the institutional review boards of the investigators; the research protocol has undergone ethical review approval (00402113712112520230227). Therefore, the project will comply with ethical research principles, including the issues of confidentiality, legality, professionalism, consent, privacy, and protection from harm. This research protocol has also been prospectively registered in the UMIN Clinical Trial Registry (UMIN000051386) on June 20, 2023.

## Results

By adopting the intention-to-treat principle, there will be significant mean changes in GAD scores and FGID scores after exposure to this combined approach in the Indonesian and South African populations. Implementing this comprehensive intervention will improve the students’ psychological symptoms related to FGIDs and ultimately enhance their overall well-being.

## Discussion

### Primary Findings

This study will aim to develop and implement a model of counseling intervention for female students with GAD obtained from FGIDs using a combination approach of CBT, web-based counseling, and local wisdom in Indonesian and South African populations. A substantial proportion of the South African population consists of adolescents, representing 17.4% of the population and accounting for 2.1% of the overall mortality rate [54]. Meanwhile, it is estimated that the Indonesian population consists of 46 million adolescents; this makes it vital for the nation to promote adolescents’ health and well-being to fully take advantage of its demographic strength [55]. Adolescents are regarded as the potential workforce and contributors to national development and socioeconomic growth, which is why their health and well-being are paramount as they form a cornerstone of society [54]. However, the adolescent population may face some mental health challenges, such as GAD. Anxiety experienced by individuals (in this case, students) is closely related to the emergence of various health problems, especially digestive conditions [8,22-24]. This anxiety condition becomes psychopathological, which has a reasonably close and reciprocal correlation with the emergence of various GI problems. The problems most commonly experienced by students who experience anxiety are FGID symptoms, which are a group of disorders characterized by chronic GI symptoms (eg, abdominal pain, dysphagia, dyspepsia, diarrhea, constipation, and bloating) without any pathology that can be proven in conventional testing [25-27]. When individuals experience FGIDs, they tend to also experience anxiety reactions. This is so recurrent that it is challenging to stop the cycle of problems between psychological and physical disorders[28,29].

Previous studies have also revealed the relationship between psychological problems (especially anxiety) and FGIDs. This condition tends to be experienced in the form of dyspeptic disorders, with a correlation prevalence of 0.48 [30,31]. In addition, other studies show a strong relationship between FGIDs and major depressive episodes and GADs [56]. As a form of reciprocity, studies have shown that the incidence of psychological disorders in patients with FGIDs is significantly higher than in non-FGID patients; symptoms of FGIDs are strongly associated with mental health disorders, especially anxiety and depression [49,50]. Pathophysiological studies of FGIDs have shown that psychosocial factors can influence FGIDs by regulating processing pathways and decreasing visceral signaling in the brain [29,32]. Analyses of interventions for irritable bowel syndrome have shown that psychological interventions, including web-based CBT, could treat irritable bowel syndrome effectively in patients [56,57].

This study hypothesizes that there will be significant mean changes in GAD scores and FGID scores after exposure to this combined approach of CBT, web-based counseling, and local wisdom in the Indonesian and South African populations. So far, pharmacotherapy and psychological interventions have undertaken the management of anxiety that results in FGIDs, and vice versa [25,28]. These psychological interventions include psychotherapy, psychodrama, CBT, relaxation therapy, and hypnosis [33-36]. Psychological intervention refers to psychotherapeutic methods designed to change a person’s cognition, perception, or behavior. Psychodynamic psychotherapy focuses on how maladaptive thoughts and behaviors occur [35,36]. The psychodynamics of interpersonal psychotherapy are more concerned with the relationship between therapist and patient. This method emphasizes that the therapist and patient form a robust, cooperative working alliance. Meanwhile, CBT aims to improve the quality of life by changing the patient’s thoughts or patterns of thinking and behavior. Relaxation therapy allows the patient to experience the physical and mental pleasure that relaxation brings to correct the psychological and physiological dysfunction caused by tension [12,37,38]. Hypnotherapy is the use of hypnosis to treat patients and improve their condition. For this reason, it is necessary to have a new approach that uses collaborative and cross-approach concepts to get more effective and efficient results. Following previous research, there are empirical results regarding the impact of psychological interventions on reducing anxiety symptoms and improving pain due to FGID; there is still no single fixed model with a combination of approaches to reduce anxiety conditions effectively. In addition, existing research has not shown an in-depth evaluation process of research results in the adolescent population. This study uses a CBT approach that has proven useful for clients with maladaptive disorders such as stress and anxiety. The study also uses the web-based counseling intervention approach as a way to evaluate and supervise the progress of counseling and also uses the implementation of local wisdom. The therapeutic model of local wisdom is added to this study, considering that behavioral change is closely related to the cultural values it carries [39-41]. In this respect, the Ubuntu counseling model as a local wisdom approach will be integrated to assist the South African adolescents during the treatment sessions since it involves integrating African culture into the therapeutic process in that it recognizes the relevance of culture to patient mental health and self-identity [58]. This study uses cross-cultural analysis with very different cultures, namely Indonesian and South African cultures. The cross-cultural analysis will show whether the intervention can be applied to 2 different cultures with almost the same indications of anxiety in terms of symptoms and aggravating factors. The results of this study will contribute to the literature on GAD and FGID treatment by informing clinicians about the potential benefits of CBT combined with local wisdom and web-based counseling approaches for adolescent girls with FGIDs experiencing GAD. However, in this study, there are some potential limitations, including that the treatment pattern does not allow for the blinding of therapists, the possibility that participants may use other interventions or treatments while taking part in the study, and a sample size that may make it impossible to generalize the study results to a larger population and spot subtler effects. While participating in the trial, participants will be required to speak frankly about their medications and treatments. In addition, they will be required to notify the researchers about any modifications to their medication doses throughout the study. In this way, the results can be taken into account with respect to any confounding variables.

### Conclusions

This study will develop a model counseling intervention for female students with GAD caused by FGID using a combination approach to CBT, web-based counseling, and local wisdom in Indonesian and South African populations. The study proposes that there will be significant mean changes in GAD scores and FGID scores after exposure to this combined approach of CBT, web-based counseling, and local wisdom in the Indonesian and South African populations. The use of drugs, especially benzodiazepines, often causes side effects on psychological conditions. Thus, it is necessary to have a solution in the form of a targeted and efficient approach to reduce psychological symptoms that arise from FGID in the form of anxiety. With a specific target of anxiety sufferers in the female population of student age, it is deemed appropriate to provide a CBT approach combined with the implementation of web-based counseling and local wisdom. The trial findings will contribute to our understanding of the effects of CBT combined with local wisdom and web-based counseling approaches that mental health counselors can use to treat GAD-affected adolescent girls with FGIDs.

## Appendix

Multimedia Appendix 1 Peer-review by the World Class University International Collaborative Research Program, Universitas Negeri Padang.

## Abbreviations

CACE: complier-adjusted causal effect
CBT: cognitive behavioral therapy
CONSORT: Consolidated Standards of Reporting Trials
FGI: functional gastrointestinal
FGI-Checklist: Functional Gastrointestinal Disorder Checklist
FGID: functional gastrointestinal disorder
GAD: generalized anxiety disorder
GAD-7: Generalized Anxiety Disorder 7-item
GI: gastrointestinal
LMM: linear mixed model

## Data Availability

The data sets generated during or analyzed during this study will be made available from the corresponding author on reasonable request.
