# Supplementary material for: Efficacy of Cognitive Behavioral Therapy With Local Wisdom and Web-Based Counseling on Generalized Anxiety Disorders and Functional Gastrointestinal Disorders in Adolescent College Girls: Protocol for a Randomized Controlled Trial
Source: JMIR Res Protoc. 2023 Aug 22;12:e50316. doi: 10.2196/50316 (PMC10481218; doi:10.2196/50316)
Supplement: Multimedia Appendix 1 [file resprot_v12i1e50316_app1.pdf]

KEMENTERIAN PENDIDIKAN, KEBUDAYAAN, RISET DAN TEKNOLOGI
UNIVERSITAS NEGERI PADANG
Address :Jln. Prof. Dr. Hamka Air Tawar Padang 25131 Tel: 0751-7053902 Fax:0751-7055628
Website: http://www.unp.ac.id e-mail: info@unp.ac.id

NOTULENSI RAPAT PIMPINAN LEMBAGA PENELITIAN DAN PENGABDIAN KEPADA MASYARAKAT UNIVERSITAS NEGERI PADANG

Agenda : Penentuan Kesimpulan Hasil Review terhadap Usulan Penelitian
Jadwal : 15 November 2022

Judul Usulan: *Pengembangan Model Intervensi Konseling Pada Remaja Wanita Dengan Kondisi Functional Gastrointestinal Disorders (FGID) Serta Generalized Anxiety Disorder (GAD) Berbasis Pendekatan Cognitive Behavior Therapy, Konseling Online, Dan Kearifan Lokal: Studi Cross-Cultural Pada Mahasiswa Indonesia Dan Afrika Selatan*

Pengusul: Dr. Zadrian Ardi, M.Pd., Kons.

Anggota yang hadir:
- Prof. Yohandri, M.Si, Ph.D (*Pimpinan Rapat/Perwakilan Grantor*)
- Prof. Dr. Anton Komaini, S.Si., M.Pd.
- Prof. Dr. Rahadian Z, S.Pd, M.Si.
- Prof. Ifdil, S.HI., S.Pd., M.Pd., Ph.D., Kons
- Dr. Ir. Krismadinata, S.T., M.T.

<u>KEGIATAN</u>
1. Pimpinan rapat membuka kegiatan dengan resmi
2. Sekretaris lembaga (Prof. Dr. Anton Komaini, S.Si., M.Pd) memberikan paparan mengenai rincian hibah penelitian, luaran dan aktivitas yang harus dilakukan penerima hibah.
3. Kepala pusat penelitian (Dr. Ir. Krismadinata, S.T., M.T.) membacakan hasil review terkait usulan dari pengusul (**lihat APPENDIX 1**).
4. Dewan mendiskusikan substansi review, kemungkinan pendanaan dan hasil perbaikan yang telah dilakukan oleh pengusul pada proposal (**lihat APPENDIX 2**)
5. Dewan memutuskan untuk memberikan dan menyetujui hibah penelitian Kerjasama Internasional WCU kepada pengusul dengan nomor Kontrak 4938/UN35.13/KP/2022 sebagaimana pada **APPENDIX 3** dan dapat diumumkan melalui SK Rektor UNP.
6. Pengusul terlebih dahulu harus memperbaiki usulan sesuai dengan masukan reviewer
7. Rapat ditutup pimpinan

KESIMPULAN
Usulan <u>DIDANAI (FUNDED)</u>

Padang, 16 November 2022
Kepala LPPM UNP

Prof. Yohandri, M.Si, Ph.D

Appendix 1

PROPOSAL BLIND REVIEW

Reviewer 1
Reviewer Number : 0025026106
Reviewer Code : FM01

KOMENTAR DAN REKOMENDASI ATAS PROPOSAL
COMMENTS AND RECOMMENDATIONS ON THE PROPOSAL

Novelty, Rumusan Masalah dan Urgensi Riset
Novelty, Problem Formulation and Research Urgency

Novelty riset belum tergambar dengan jelas dalam naskah proposal ini. Meskipun disajikan dengan beberapa referensi penelitian sebelumnya, tidak ada penjelasan yang cukup mengenai bagaimana riset ini akan memberikan kontribusi baru dalam pemahaman kita tentang gangguan FGID dan kecemasan pada populasi wanita mahasiswa. Diperlukan penjelasan lebih lanjut tentang bagaimana pendekatan Cognitive Behavior Therapy (CBT) yang dikombinasikan dengan implementasi Konseling Online dan kearifan lokal akan menjadi solusi yang inovatif dan efektif dalam mengurangi gejala psikologis yang terkait dengan gangguan FGID.
Rumusan masalah dalam naskah proposal ini juga masih perlu ditekankan secara eksplisit. Meskipun diidentifikasi bahwa gangguan FGID dan kecemasan memiliki dampak negatif pada kualitas hidup dan situasi mental mahasiswa, tidak ada pernyataan yang jelas tentang masalah penelitian yang akan diteliti dan dipecahkan oleh riset ini. Penting untuk merumuskan secara jelas pertanyaan penelitian dan tujuan penelitian yang spesifik agar tujuan riset menjadi lebih terfokus.
Selain itu, justifikasi urgensi dari riset ini juga perlu diperkuat. Meskipun disebutkan bahwa upaya-upaya sebelumnya belum memberikan hasil yang signifikan, tidak ada penjelasan yang memadai tentang mengapa pendekatan yang diusulkan (CBT dikombinasikan dengan implementasi Konseling Online dan kearifan lokal) dianggap sebagai solusi yang tepat dan efisien. Diperlukan penjelasan lebih lanjut tentang bagaimana pendekatan ini diharapkan dapat memberikan perubahan positif pada mahasiswa wanita dengan kondisi FGID dan kecemasan.

The novelty of the research is not clearly portrayed in this proposal. Although it is presented with several previous research references, there is not enough explanation about how this research will contribute new insights into our understanding of FGID and anxiety disorders among female student populations. Further clarification is needed on how the combination of Cognitive Behavior Therapy (CBT) with the implementation of Online Counseling and local wisdom will offer an innovative and effective solution in reducing psychological symptoms related to FGID. The formulation of the research problem in this proposal also needs to be explicitly emphasized. Despite the identification of the negative impacts of FGID and anxiety on students' quality of life and mental well-being, there is no clear statement regarding the

specific research problem to be investigated and addressed by this study. It is important to formulate research questions and objectives that are specific and focused to ensure the research goals are well-defined. Additionally, the justification for the urgency of this research needs to be strengthened. Although it is mentioned that previous efforts have not yielded significant results, there is inadequate explanation as to why the proposed approach (CBT combined with Online Counseling and local wisdom) is considered an appropriate and efficient solution. Further elaboration is needed on how this approach is expected to bring about positive changes for female students with FGID and anxiety conditions.

Metodologi dan Penggunaan Dana
Methodology and Use of Funds

Metodologi yang dijelaskan dalam naskah proposal ini masih terlalu umum dan perlu untuk diperinci. Tahapan riset yang disajikan perlu diperinci dengan lebih jelas, terutama dalam konteks penggunaan dana yang diusulkan. Penjelasan yang lebih rinci tentang bagaimana dana akan digunakan dalam setiap tahap riset akan membantu memperkuat rencana penelitian dan memastikan penggunaan dana yang efisien.
Selain itu, perlu disebutkan dengan lebih jelas peran setiap anggota tim riset dalam pelaksanaan penelitian. Deskripsi yang lebih terperinci tentang tanggung jawab dan kontribusi masing-masing anggota tim akan membantu memperjelas alur kerja dan memastikan kerjasama yang efektif dalam tim.
Alur penelitian atau alur ADDIE yang diadopsi perlu diperjelas dengan lebih terinci, termasuk integrasi dengan luaran penelitian yang diharapkan. Jelaskan bagaimana setiap tahap penelitian akan menghasilkan luaran khusus dan bagaimana luaran tersebut akan diintegrasikan untuk mencapai tujuan penelitian yang telah ditetapkan. Hal ini akan membantu membentuk gambaran yang lebih jelas tentang bagaimana penelitian akan dilaksanakan dan bagaimana hasilnya akan digunakan atau berkontribusi dalam konteks yang lebih luas.
Dalam tahap uji coba kelompok kecil dan uji coba kelompok terbatas, perlu disebutkan dengan lebih jelas mengenai metode yang akan digunakan untuk mengumpulkan data, seperti metode pengumpulan data kualitatif dan eksperimen. Jelaskan juga bagaimana data yang terkumpul akan dievaluasi dan digunakan untuk merevisi model yang dikembangkan.
Secara keseluruhan, metodologi yang dijelaskan dalam naskah proposal ini perlu diperinci dan disesuaikan dengan dana yang diusulkan. Peran setiap anggota riset harus tampak dan alur penelitian harus diperjelas dengan integrasi yang lebih baik dengan luaran penelitian. Dengan perbaikan ini, metodologi penelitian akan menjadi lebih solid dan terperinci.

The methodology described in this proposal is still too general and needs to be further detailed. The research stages presented need to be clarified more explicitly, especially in the context of the proposed funding usage. Providing more detailed explanations of how the funding will be utilized in each research stage will strengthen the research plan and ensure efficient use of the funds.
Furthermore, it is necessary to clearly mention the roles of each research team member in the implementation of the study. A more detailed description of the responsibilities and contributions of each team member will help clarify the workflow and ensure effective collaboration within the team.

The research flow or ADDIE process adopted needs to be further elaborated, including the integration with the expected research outcomes. Explain how each research stage will produce specific outcomes and how these outcomes will be integrated to achieve the predetermined research objectives. This will help form a clearer picture of how the research will be conducted and how the results will be utilized or contribute in a broader context.
In the stages of small-scale testing and limited-group testing, it is important to provide clearer information about the methods that will be used to collect data, such as qualitative data collection methods and experiments. Also, explain how the collected data will be evaluated and used to revise the developed model.
Overall, the methodology described in this proposal needs to be further detailed and aligned with the proposed funding. The roles of each research team member should be apparent, and the research flow should be clarified with better integration with the research outcomes. With these improvements, the research methodology will become more solid and detailed.

Kekuatan Proposal
Proposal Strength

Kekuatan usulan proposal ini terletak pada identifikasi masalah yang relevan, yaitu gangguan Functional Gastrointestinal Disorders (FGID) dan kecemasan pada mahasiswa wanita. Pendekatan yang diusulkan, yaitu menggabungkan Cognitive Behavior Therapy (CBT) dengan implementasi Konseling Online dan kearifan lokal, menunjukkan upaya untuk mengatasi masalah ini secara holistik.
Selain itu, penggunaan metode pengembangan berbasis model ADDIE memberikan kerangka kerja yang sistematis dan terstruktur untuk mengembangkan dan menguji model asesmen. Penggunaan studi literatur dan analisis awal untuk merancang model asesmen juga merupakan langkah yang baik dalam membangun dasar yang kuat untuk penelitian ini.
Usulan untuk melibatkan ahli dan kelompok sasaran dalam tahap pengembangan dan pengujian model menunjukkan keinginan untuk menghasilkan solusi yang relevan dan efektif. Pengujian pada kelompok kecil dan kelompok terbatas serta penggunaan metode eksperimen memberikan kesempatan untuk menguji dan mengukur dampak dari penggunaan model yang dikembangkan.
Kombinasi antara pendekatan terapi psikologis (CBT) dengan pemanfaatan teknologi (Konseling Online) dan pengintegrasian kearifan lokal juga menunjukkan upaya untuk menghadirkan solusi yang praktis dan relevan dengan konteks mahasiswa wanita.
Secara keseluruhan, kekuatan usulan proposal ini terletak pada identifikasi masalah yang relevan, pendekatan holistik yang diusulkan, penggunaan metode pengembangan yang terstruktur, serta upaya untuk melibatkan ahli dan kelompok sasaran dalam pengembangan dan pengujian model.

The strength of this proposal lies in the identification of relevant issues, namely Functional Gastrointestinal Disorders (FGID) and anxiety among female students. The proposed approach, which combines Cognitive Behavior Therapy (CBT) with the implementation of Online Counseling and local wisdom, demonstrates an effort to address these problems holistically.
Additionally, the use of the ADDIE-based development method provides a systematic and structured framework for developing and testing the assessment model. The utilization of

literature studies and initial analysis to design the assessment model is also a good step in establishing a strong foundation for this research.
The proposal's suggestion to involve experts and target groups in the development and testing stages of the model shows a desire to generate relevant and effective solutions. Testing with small-scale and limited groups, as well as the use of experimental methods, provides an opportunity to assess and measure the impact of the developed model.
The combination of psychological therapy (CBT) with the utilization of technology (Online Counseling) and the integration of local wisdom also demonstrates an effort to provide practical and contextually relevant solutions for female students.
Overall, the strengths of this proposal lie in the identification of relevant issues, the proposed holistic approach, the use of structured development methods, and the effort to involve experts and target groups in the development and testing of the model.

Kelemahan Proposal
Proposal Weakness

Usulan proposal ini memiliki kelemahan dalam penjelasan tentang kontribusi baru yang akan diberikan oleh penelitian ini, rumusan masalah yang masih perlu diperinci, serta justifikasi urgensi yang belum diperkuat secara memadai. Pada proposal tidak ditemukan adanya jadwal kegiatan penelitian, Tidak diperkenankan untuk pembelian barang inventaris.

This proposal has weaknesses in explaining the new contributions that will be provided by this research, the formulation of the research problem that still needs further elaboration, and the inadequate justification of the research urgency. The proposal also lacks a research activity schedule. Additionally, it is not allowed to purchase inventory items.

Kesimpulan Review
Review Conclusion

Rumusan masalah penelitian dan Tujuan penelitian hendaknya dibuat dengan jelas dan rinci; Desain penelitian yang digunakan harus jelas dan harus taat azas dengan desain yang digunakan; Pedomani Peraturan Menteri Keuangan (PMK) tentang Standar Biaya Keluaran dan Peraturan Menteri Keuangan (PMK) RI tentang Standar Biaya Masukan Tahun Anggaran yang sedang berjalan; ikuti format penulisan yang ada; Proposal dirancang untuk 1 tahun penelitian (tidak multi tahun). ***Secara umum proposal dapat diterima dengan perbaikan.***

The research problem formulation and research objectives should be clear and detailed. The research design used must be clearly stated and adhere to the principles of the chosen design. It is important to follow the regulations of the Ministry of Finance regarding the Standard Cost of Outputs and the Standard Cost of Inputs for the current fiscal year. Additionally, adhere to the existing writing format. The proposal is designed for a one-year research project (not multi-year). ***In general, the proposal can be accepted with improvements.***

Evaluasi Kuantitatif

Evaluation

5=Excellent 4=Good 3=Average 2=Below Average 1=Poor n/a=Not Applicable

Items	Grade
Novelty of Research	4
Contribution to existing knowledge	4.2
Soundness of methodology	3.5
Appropriate formatting and structure	4.5
Readability	4.5
Evidence supports conclusion	4.5
Adequacy of literature review	5

Rekomendasi Pendanaan
Funding Recommendations

DIDANAI
FUNDED

:: End of Review ::

KEMENTERIAN PENDIDIKAN, KEBUDAYAAN, RISET DAN TEKNOLOGI
UNIVERSITAS NEGERI PADANG
Address :Jln. Prof. Dr. Hamka Air Tawar Padang 25131 Tel: 0751-7053902 Fax:0751-7055628
Website: http://www.unp.ac.id e-mail: info@unp.ac.id

PROPOSAL BLIND REVIEW

Reviewer 2
Reviewer Number : 0011128104
Reviewer Code : IFD08

KOMENTAR DAN REKOMENDASI ATAS PROPOSAL
COMMENTS AND RECOMMENDATIONS ON THE PROPOSAL

Novelty, Rumusan Masalah dan Urgensi Riset
Novelty, Problem Formulation and Research Urgency

Novelty dalam usulan proposal ini dapat dipahami dengan baik karena mengidentifikasi masalah yang relevan, yaitu gangguan Functional Gastrointestinal Disorders (FGID) dan kecemasan pada mahasiswa wanita, serta mencoba menggabungkan pendekatan Cognitive Behavior Therapy (CBT) dengan implementasi Konseling Online dan kearifan lokal. Namun, untuk lebih memperjelas novelty tersebut, proposal ini perlu menyertakan pernyataan yang lebih eksplisit tentang poin-poin khusus yang akan dikembangkan. Pernyataan tersebut dapat mencakup kontribusi baru yang akan diberikan dalam pemahaman tentang FGID dan kecemasan, atau inovasi dalam penggunaan kombinasi pendekatan yang diusulkan. Dengan demikian, pernyataan yang lebih spesifik tentang novelty akan membantu memperkuat usulan proposal ini.

The novelty of this proposal can be understood well as it identifies relevant issues, such as Functional Gastrointestinal Disorders (FGID) and anxiety among female students, and attempts to combine Cognitive Behavior Therapy (CBT) with the implementation of Online Counseling and local wisdom. However, to further clarify the novelty, this proposal needs to include more explicit statements about the specific points that will be developed. These statements can encompass the new contributions that will be made in understanding FGID and anxiety, or the innovations in the proposed combination of approaches. Therefore, more specific statements about the novelty will help strengthen this proposal

Metodologi dan Penggunaan Dana
Methodology and Use of Funds

Metodologi sudah jelas dan dapat dieksekusi dengan baik.

The methodology is clear and can be executed well.

Kekuatan Proposal
Proposal Strength

Kekuatan proposal terletak pada kejelasan dan keterlaksanaan metodologi. Metodologi yang jelas dan dapat dieksekusi dengan baik sangat penting untuk menjalankan penelitian yang efektif. Hal ini menunjukkan bahwa para peneliti telah memikirkan proses penelitian dengan baik dan memiliki rencana yang kokoh untuk melaksanakan studi tersebut. Metodologi yang terdefinisi dengan baik meningkatkan kredibilitas proposal dan meningkatkan kemungkinan untuk mendapatkan hasil yang dapat diandalkan dan valid. Oleh karena itu, kekuatan proposal dalam hal metodologi adalah aspek positif yang berkontribusi pada kualitas keseluruhan proposal.

The strength of the proposal lies in the clarity and feasibility of the methodology. A clear and well-executed methodology is crucial for conducting effective research. It indicates that the researchers have thought through the research process and have a solid plan for carrying out the study. A well-defined methodology enhances the credibility of the proposal and increases the likelihood of obtaining reliable and valid results. Therefore, the strength of the proposal in terms of its methodology is a positive aspect that contributes to its overall quality.

Kelemahan Proposal
Proposal Weakness

Kelemahan proposal adalah kurangnya pernyataan eksplisit tentang poin-poin khusus yang akan dikembangkan untuk memperjelas novelty riset. Proposal perlu memberikan penjelasan yang lebih rinci mengenai kontribusi baru yang akan diberikan dalam pemahaman tentang gangguan FGID dan kecemasan, serta inovasi yang diusulkan dalam penggunaan kombinasi pendekatan yang diajukan. Dengan memperkuat pernyataan tentang novelty secara spesifik, proposal dapat menjadi lebih kuat dan memberikan gambaran yang lebih jelas tentang kontribusi penelitian ini terhadap bidang yang dipelajari.

The weakness of the proposal lies in the lack of explicit statements regarding the specific points that will be developed to clarify the novelty of the research. The proposal needs to provide more detailed explanations about the new contributions that will be made in understanding FGID and anxiety, as well as the proposed innovations in the utilization of the combined approaches. By strengthening the statements about the novelty in a more specific manner, the proposal can become stronger and provide a clearer overview of the research's contribution to the field.

Kesimpulan Review
Review Conclusion

Penelitian yang akan dilakukan jelas, kelengkapan proposal lengkap serta berpotensi ketercapaian luaran. ***Secara umum proposal dapat diterima untuk didanai.***

The research to be carried out is clear, the completeness of the proposal is complete and has the potential for achieving outcomes. ***In general, proposals are acceptable for funding.***

Evaluasi Kuantitatif

Evaluation

5=Excellent 4=Good 3=Average 2=Below Average 1=Poor n/a=Not Applicable

Items	Grade
Novelty of Research	4.5
Contribution to existing knowledge	5
Soundness of methodology	4
Appropriate formatting and structure	5
Readability	4.5
Evidence supports conclusion	5
Adequacy of literature review	5

Rekomendasi Pendanaan
Funding Recommendations

DIDANAI
FUNDED

:: End of Review ::

KEMENTERIAN PENDIDIKAN, KEBUDAYAAN RISET, DAN TEKNOLOGI
UNIVERSITAS NEGERI PADANG
Jln. Prof. Dr. Hamka Air Tawar Padang 25131
Telepone : 0751-7053902 Fax:0751-7055628
Website: http://www.unp.ac.id e-mail: rektor@unp.ac.id

LETTER OF RESEARCH GRANT NOTIFICATION
INTERNATIONAL RESEARCH COLLABORATION
WORLD CLASS UNIVERSITY
UNIVERSITAS NEGERI PADANG

Based on the decision of the Rector of Universitas Negeri Padang, Indonesia, with Number 1212/UN35.13/KP/2022, it can be noted that research in detail:

Research Title	: **Development of a Counseling Intervention Model for Adolescent Women with Functional Gastrointestinal 3 Disorders (FGID) and Generalized Anxiety Disorder (GAD) based on Cognitive Behavior Therapy Approaches, Online Counseling, and Local Wisdom: A Cross-Cultural Study of Indonesian and South African Students**
Research Duration	: 1 Year
Research Team	: 1. Dr. Zadrian Ardi, M.Pd., Kons. (Universitas Negeri Padang, Indonesia)<br>2. Dr. Chiedu Eseadi (University of Johannesburg, South Africa)<br>3. Dr. dr. Elsa Yuniarti, S.ked., M.Biomed. (Universitas Negeri Padang, Indonesia)<br>4. Frischa Meivilona Y, M.Pd., Kons. (Universitas Negeri Padang, Indonesia)

Type of Grant : Package B

Declared to have passed the assessment and received funding based on the World Class University International Collaborative Research Program from Universitas Negeri Padang. The list of research grant winners is attached.

All costs incurred from this program will be charged to the Higher Education Endowment Fund program with Number 2833/E3/KB.07.02/2022 and Number 2056/UN35.TU/2022.

Thus this statement letter is notified so that it can be used properly.

Padang, 29 November 2022
Rector,

KEMENTERIAN PENDIDIKAN, KEBUDAYAAN, RISET DAN TEKNOLOGI UNIVERSITAS NEGERI PADANG REKTOR

Prof. Ganefri, Ph,D
NIP.196312171989031003

Appendix 2
FINAL PROPOSAL

PROPOSAL
HIBAH RISET KOLABORASI INTERNASIONAL WCU
WORLD CLASS UNIVERSITY (WCU)

Pengembangan Model Intervensi Konseling pada Remaja Wanita dengan kondisi Functional Gastrointestinal Disorders (FGID) serta Generalized Anxiety Disorder (GAD) berbasis Pendekatan Cognitive Behavior Therapy, Konseling Online, dan Kearifan Lokal: Studi Cross-Cultural pada Mahasiswa Indonesia dan Afrika Selatan

Oleh:

**Dr. Zadrian Ardi, M.Pd., Kons.**	**NIDN: 0001069001 (Ketua Tim Pengusul)**
**Dr. Chiedu Eseadi**	**University of Johannesburg, South Africa**
**Dr. dr. Elsa Yuniarti, S.ked., M.Biomed., AIFO-K**	**NIDN: 0023068204 (Anggota Tim Pengusul)**
**Frischa Meivilona Y, M.Pd., Kons**	**NIDN:0021049101 (Anggota Tim Pengusul)**

FAKULTAS ILMU PENDIDIKAN
UNIVERSITAS NEGERI PADANG
2022

HALAMAN PENGESAHAN

Judul	: Pengembangan Model Intervensi Konseling pada Remaja Wanita dengan kondisi Functional Gastrointestinal Disorders (FGID) serta Generalized Anxiety Disorder (GAD) berbasis Pendekatan Cognitive Behavior Therapy, Konseling Online, dan Kearifan Lokal: Studi Cross-Cultural pada Mahasiswa Indonesia dan Afrika Selatan
Fokus Riset UNP	: Media dan Sumber Belajar di ERI 4.0
Target SDG's	: Good Health and Well-being, Quality Education
Skema	: Hibah Riset Kolaborasi Internasional WCU
Rumpun Ilmu	: Ilmu Pendidikan
Fakultas	: Fakultas Ilmu Pendidikan
Universitas Kolaborator	: University of Johannesburg, South Africa (QS 301 by Subject Education and Training)

Pengusul

a. Nama Lengkap	: Dr. Zadrian Ardi, M.Pd., Kons.
b. NIP/NIDN	: 199006012015041002 / 0001069001
c. Jabatan Fungsional	: Lektor
d. Program Studi	: Bimbingan dan Konseling
e. Nomor HP	: 085274030264
f. Alamat surel (e-mail)	: zadrian@fip.unp.ac.id
g. Total biaya yang diusulkan	: Rp.100.000.000,-

Padang, 21 September 2022

**Menyetujui,**	
**Wakil Dekan 1 FIP UNP**	**Periset Utama**
**Prof. Dr. Hadiyanto, M.Ed**	**Dr. Zadrian Ardi, M.Pd., Kons.**
**NIP. 196004161986031004**	**NIP.199006012015041002**

KEMENTERIAN PENDIDIKAN, KEBUDAYAAN, RISET, DAN TEKNOLOGI UNIVERSITAS NEGERI PADANG FAKULTAS ILMU PENDIDIKAN

Abstrak

Tingginya tuntutan akademik yang dialami mahasiswa berdampak pada kualitas kesehatan mental maupun fisik mahasiswa. Permasalahan kesehatan yang paling sering muncul adalah gangguan gastrointestinal dengan prevalensi 40.85% pada rentang usia 18-25 tahun. Kondisi ini cenderung berefek pada munculnya gangguan kecemasan menyeluruh dan menurunkan kualitas hidup serta produktivitas. Populasi yang paling banyak menderita gangguan ini adalah remaja wanita, dan terjadi baik pada populasi Afrika Selatan maupun Indonesia. Berbagai upaya untuk mengentaskan permasalahan ini telah dilakukan namun tidak dapat memberikan hasil yang signifikan. Antara lain dengan menggunakan pendekatan farmakoterapi untuk meringankan gejala/symtomps kecemasan yang muncul. Untuk itu, perlu adanya solusi berupa pendekatan intervensi konseling yang tepat sasaran dan efisien untuk mengurangi gejala psikologis yang muncul dari gangguan Functional Gastrointestinal Disorders (FGID) dalam bentuk kecemasan (anxiety). Dengan sasaran khusus pada penderita kecemasan pada populasi wanita dengan usia mahasiswa, maka dirasa tepat untuk memberikan pendekatan Cognitive Behavior Therapy (CBT) dikombinasikan dengan implementasi Konseling Online dan aspek kearifan lokal. Penelitian ini diselenggarakan selama 3 (tiga) tahun dan bertujuan untuk mengembangkan model intervensi konseling untuk mahasiswa wanita dengan kondisi Generalized Anxiety Disorders (GAD) yang didapatkan dari gangguan FGID dengan menggunakan pendekatan kombinasi Cognitive Behavior Therapy (CBT), Konseling Online dan Kearifan Lokal pada populasi Indonesia dan Afrika Selatan. Subjek penelitian terdiri dari mahasiswa wanita dari Indonesia sebanyak 500 orang dan Afrika Selatan sebanyak 500 orang. Luaran penelitian ini adalah publikasi pada International Journal of Psychological Research Q3 (SJR 0.28) dan prosiding terindeks Scopus. Sedangkan pada tahun kedua akan dihasilkan draft/prototype model dan pada tahun ketiga akan dihasilkan final model sesuai tujuan penelitian.

1. Latar Belakang (Background)

Mahasiswa mengalami berbagai perubahan dan tantangan dalam menjalani studi di perguruan tinggi. Dengan tingginya tuntutan akademik yang harus dipenuhi oleh mahasiswa dan kecenderungan lemahnya kontrol terhadap kesehatan berdampak pada kondisi diri mahasiswa [1, 2]. Berbagai gangguan pada kesehatan mental juga cenderung meningkat pada fase individu sebagai mahasiswa [3-5]. Kondisi ini cenderung menimbulkan masalah kesehatan dan berpengaruh pada kualitas hidup dan situasi mental dari mahasiswa. Gangguan yang umum muncul pada mahasiswa adalah Functional Gastrointestinal Disorders (FGID) [6, 7]. FGID cenderung muncul ditandai dengan nyeri perut, disfagia, dispepsia, diare, sembelit, kembung dan gastrointestinal akut [7-9]. Berdasarkan data Kementerian Kesehatan Republik Indonesia tahun 2020, gangguan FGID termasuk pada 10 penyakit yang banyak diderita oleh remaja hingga dewasa muda di Indonesia dengan prevalensi hingga 40.85% dan rentang usia 18 – 25 tahun [10, 11]. Sedangkan pada populasi Afrika Selatan, terdapat 8% dari total populasi mengalami hal ini, seperti di provinsi KwaZulu-Natal Afrika Selatan, ditemukan bahwa di antara 201 pasien pedesaan yang disajikan dengan salah satu jenis FGID (dispepsia fungsional), gangguan kecemasan dilaporkan oleh sebagian besar pasien (74,13%) [12]. Individu dengan gangguan FGID cenderung mengalami gangguan kecemasan, sebaliknya indivud dengan kondisi kecemasan yang dominan akan mengalami gangguan pada pencernaannya [8, 13-15]. Namun, penanganan gangguan FGID berdasarkan temuan terbaru umumnya masih mengandalkan terapi obat-obatan. Dimana terapi ini cenderung menghasilkan efek samping lain yang diderita oleh pasien [16]. Selain itu, pengobatan FGID yang pada hakikatnya tidak memunculkan kondisi organik justru menghabiskan banyak waktu dan biaya. Selain itu, kondisi kecemasan dari pasien tidak kunjung mereda dan cenderung memburuk [14, 17]. Gangguan kecemasan sebagai dampak dari FGID ini sering dialami oleh populasi wanita (prevalensi Indonesia berada pada 67.8% [11]), sedangkan pada wanita di Afrika Selatan memiliki prevalensi 70.3% serta umumnya berada pada usia produktif (18-30 tahun) [18, 19]. **Dampaknya** jika kondisi kecemasan dan gangguan FGID tidak ditangani dengan baik maka akan memberikan dampak pada penurunan produktivitas dan kualitas hidup mahasiswa. Umumnya dampak ini akan berakibat pada penurunan kesemapatan mahasiswa dalam berprestasi, penurunan hasil belajar hingga berujung pada tidak dapat melanjutkan studi [9, 14, 17, 20]. **Untuk itu, perlu adanya solusi** berupa model intervensi konseling yang tepat sasaran dan efisien untuk mengurangi gejala psikologis yang muncul dari gangguan FGID berupa kecemasan (anxiety). Dengan sasaran khusus pada penderita kecemasan pada populasi wanita dengan usia mahasiswa, maka dirasa tepat untuk memberikan pendekatan Cognitive Behavior Therapy (CBT) dikombinasikan dengan implementasi Konseling Online dan kearifan lokal. Kelebihan dari pendekatan ini adalah proses asesmen yang dapat dilakukan dalam skema jarak jauh dengan menggunakan teknologi Konseling Online yang telah dikembangkan pada riset penulis sebelumnya, kemudian dikombinasikan dengan pendekatan yang telah terbukti efektif dalam

melakukan terapi perubahan perilaku dan penekanan implementasi pada kearifan lokal. Selain itu, kombinasi dari ketiga unsur ini akan dapat memacu perubahan positif pada kecemasan yang dialami mahasiswa wanita dengan kondisi FGID.

2. Tujuan Riset

Secara umum tujuan penelitian ini adalah menghasilkan model intervensi konseling untuk mahasiswa wanita dengan kondisi kecemasan menyeluruh (Generalized Anxiety Disorders/GAD) yang didapatkan dari faktor Functional Gastrointestinal Disorders (FGID) dengan menggunakan pendekatan kombinasi Cognitive Behavior Therapy (CBT), Konseling Online dan Kearifan Lokal pada populasi Indonesia dan Afrika Selatan. Pada tahun pertama tujuan riset adalah untuk memperoleh kondisi secara mendalam gangguan kecemasan yang dialami mahasiswa wanita di Indonesia dan Afrika Selatan dengan kondisi klinis FGID sebagai dasar pengembangan prototype model. Pada tahun kedua, tujuan riset adalah untuk mengembangkan model intervensi konsseling berbasis CBT, Konseling Online dan kearifan lokal untuk mereduksi kecemasan (GAD) pada mahasiswa wanita dengan kondisi FGID. Sedangkan pada tahun ketiga, tujuan riset adalah finalisasi model dan implementasi model pada skema penanganan GAD dengan menerapkan unsur pendekatan psikologis dan teknologi.

3. Kebaruan (Novelty)

Berbagai riset terdahulu membuktikan keterkaitan antara masalah psikologis (terutama kecemasan) dengan gangguan FGID. Kondisi ini cenderung dialami dalam bentuk gangguan dispepsia dengan prevalensi korelasi sebesar 0.48 [21, 22]. Selain itu, penelitian lain juga menunjukkan hasil bahwa jika terdapat hubungan yang kuat antara dispepsia dengan major depressive episode dan generalised anxiety disorder. Sebagai bentuk hubungan timbal balik, studi berbasis populasi menunjukkan bahwa kejadian gangguan psikologis pada pasien dengan FGID secara signifikan lebih tinggi daripada pada pasien non-FGID. Gejala FGID sangat berhubungan dengan gangguan kesehatan mental, teurtama kecemasan dan depresi. Studi patofisiologi FGID telah menunjukkan bahwa faktor psikososial dapat mempengaruhi FGID dengan mengatur jalur pemrosesan dan penurunan sinyal visceral di otak [23, 24]. Meta-analisis dari sindrom iritasi usus besar telah menunjukkan bahwa intervensi psikologis efektif dalam pengobatan sindrom iritasi usus besar (irritable bowel syndrome/IBS).

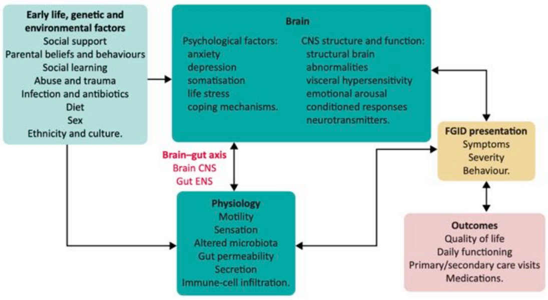

Gambar 1. Keterkaitan gangguan psikologis dengan gejala FGID dalam bentuk hubungan resiprocal.

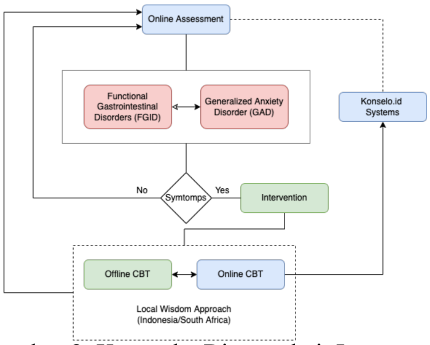

Gambar 2. Kerangka Riset terkait Intervensi Konseling dalam Mengatasi GAD pada penderita FGID

Selama ini, penanganan kecemasan yang berakibat pada FGID maupun sebaliknya dilakukan secara farmakoterapi dan didampingi dengan intervensi psikologis [25, 26]. Intervensi psikologis tersebut meliputi psikoterapi, psikodrama, Cognitive Behavior Therapy, terapi relaksasi dan hipnosis [27-30]. Intervensi psikologis mengacu pada metode psikoterapi yang dirancang untuk mengubah kognisi, persepsi, atau perilaku seseorang. Psikoterapi psikodinamik berfokus pada bagaimana pikiran dan perilaku maladaptif terjadi [29, 30]. psikodinamika psikoterapi interpersonal lebih memperhatikan hubungan antara terapis dan pasien. Metode ini menekankan bahwa terapis dan pasien membentuk aliansi kerja kooperatif yang kuat. Tujuan terapi perilaku kognitif adalah untuk meningkatkan kualitas hidup dengan mengubah pikiran atau pola pikir dan perilaku pasien. Sedangkan terapi relaksasi adalah untuk membuat pasien mengalami kesenangan fisik dan mental yang dibawa oleh relaksasi, sehingga dapat memperbaiki disfungsi psikologis dan fisiologis yang disebabkan oleh ketegangan [13, 31, 32]. Selama ini penggunaan berbagai bentuk terapi dan pendekatan tersebut tidak saling bersinergi dan belum pernah diuji secara bersama-sama.

Dengan demikian, **nilai kebaruan/novelty dari penelitian ini terletak pada kombinasi intervensi psikologis dengan pendekatan Cognitive Behavior Therapy yang telah terbukti mampu**

digunakan untuk klien dengan gangguan maladaptive seperti stres dan kecemasan dengan Konseling Online dan nilai Kearifan Lokal yang melekat pada proses maupun sistemnya. Kondisi kearifan lokal merupakan variabel yang ditambahkan pada penelitian ini dengan pertimbangan perubahan perilaku sangat berkaitan erat dengan nilai budaya yang dibawanya [33-35]. Dengan perbedaan budaya Afrika Selatan dan Indonesia, keunikan dari penelitian ini juga akan membuktikan apakah model yang telah dikembangkan efektif dan efisien pada dua budaya yang berbeda (*cross-cultural analysis*).

4. Metodologi (Methodology)

a. Tahapan Penelitian

Penelitian ini merupakan penelitian pengembangan (*development research*) untuk menyusun dan menemukan produk, prototype menggunakan rancangan yang sistematis dan konseptual melalui model yang diadaptasi dari ADDIE [36, 37]. Pentahapan pelaksanaan penelitian ini tergambar pada *fishbone* berikut ini:

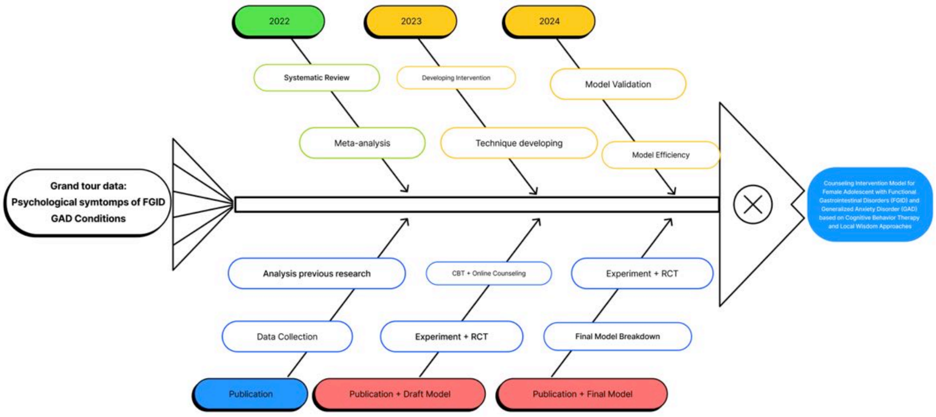

Gambar 3. Fishbone Penelitian

b. Subjek Penelitian

Sesuai dengan karakteristik dan jenis penelitian tersebut maka subjek penelitian pada tahun pertama terdiri dari mahasiswa wanita di Indonesia dan Afrika Selatan untuk proses systematic review dan analisis data sebanyak 500 orang dari masing-masing negara.

c. Teknik Pengumpulan Data

Teknik pengumpulan data yang digunakan pada penelitian ini terdiri dari:

1. Studi literatur, teknik digunakan untuk menemukan landasan teoritis dan temuan-temuan penelitian yang relevan dengan riset ini.
2. Meta-analysis berkaitan dengan teknik dan pendekatan yang efektif dalam penanganan gangguan GAD pada pasien dengan FGID sehingga dapat diterapkan pada prototype model.
3. Kuesioner, Acceptability of Mental-Health Mobile-App Survey (AMMS) digunakan untuk mengukur implementasi teknologi mobile yang ada pada model, (3) Depression Anxiety Stress Scale (DASS) untuk mengukur kondisi kecemasan yang dialami oleh responden.

d. Analisis Data

Analisis data yang digunakan dalam penelitian ini, meliputi analisis deskriptif, analisis korelasi *point biserial* untuk uji daya beda/kualitas tes dan korelasi *product moment pearson*, analisis konsistensi internal menggunakan *Alpha Cronbach* menggunakan Rasch Model Analysis, analisis *Partial Least Square* (PLS), analisis perbedaan dengan uji t untuk menguji efektivitas model.

5. Target Luaran Publikasi

Target luaran pada penelitian ini adalah:

Status Luaran	Nama Jurnal	Posisi pada SJR	Target Publikasi
Jurnal Internasional Bereputasi terindeks Scopus	International Journal of Psychological Research (https://revistas.usb.edu.co/index.php/IJPR/index)	Q3 Journal (SJR 0.28)	Accepted
Prosiding Internasional Bereputasi terindeks Scopus	The International Conference on Mathematics, Science and Technology Education (ICon-MaSTEd), Ukraina 2023 (https://icon-masted.easyscience.education/2023/)	Q3 Proceeding (IoP Publishing)	Accepted/Published

References

[1] C. A. Stamatis, H. C. Broos, S. E. Hudiburgh, S. K. Dale, and K. R. Timpano, "A longitudinal investigation of COVID-19 pandemic experiences and mental health among university students," *Br. J. Clin. Psychol.,* Article vol. 61, no. 2, pp. 385-404, 2022, doi: 10.1111/bjc.12351.

[2] R. Xu, "The Relationship Between Psychological Quality Education and Mental Health Level of College Students by Educational Psychology," *Frontiers in Psychology,* Article vol. 13, 2022, Art no. 892143, doi: 10.3389/fpsyg.2022.892143.

[3] A. Clabaugh, J. F. Duque, and L. J. Fields, "Academic stress and emotional well-being in United States college students following onset of the COVID-19 pandemic," *Frontiers in Psychology,* vol. 12, p. 628787, 2021.

[4] M. A. Karaman, E. Lerma, J. C. Vela, and J. C. Watson, "Predictors of academic stress among college students," *Journal of College Counseling,* vol. 22, no. 1, pp. 41-55, 2019.

[5] P. J. Jones, S. Y. Park, and G. T. Lefevor, "Contemporary College Student Anxiety: The Role of Academic Distress, Financial Stress, and Support," *Journal of College Counseling,* vol. 21, no. October, pp. 252-264, 2018, doi: 10.1002/jocc.12107.

[6] L. F. Damis and M. S. Hamilton, "Impact of hypnotic safety on disorders of gut-brain interaction: A pilot study," *American Journal of Clinical Hypnosis,* Article vol. 63, no. 2, pp. 150-168, 2020, doi: 10.1080/00029157.2020.1794434.

[7] C. Duan *et al.*, "Association of stress and functional gastrointestinal disorders in high school graduates," *Journal of Affective Disorders,* Article vol. 292, pp. 305-310, 2021, doi: 10.1016/j.jad.2021.05.072.

[8] X. Guo, F. Lin, F. Yang, J. Chen, W. Cai, and T. Zou, "Gut microbiome characteristics of comorbid generalized anxiety disorder and functional gastrointestinal disease: Correlation with alexithymia and personality traits," *Frontiers in Psychiatry,* vol. 13, 2022.

[9] N. Ranasinghe *et al.*, "Functional gastrointestinal diseases and psychological maladjustment, personality traits and quality of life," *BMC gastroenterology,* vol. 18, no. 1, pp. 1-10, 2018.

[10] I. Huang *et al.*, "The prevalence of uninvestigated dyspepsia and the association of physical exercise with quality of life of uninvestigated dyspepsia patients in Indonesia: An internet-based survey," *Indian Journal of Gastroenterology,* Article vol. 40, no. 2, pp. 176-182, 2021, doi: 10.1007/s12664-020-01113-z.

[11] D. N. Sari, A. W. Murni, and E. Edison, "Hubungan ansietas dan depresi dengan derajat dispepsia fungsional di RSUP Dr M Djamil Padang periode Agustus 2013 hingga Januari 2014," *Jurnal Kesehatan Andalas,* vol. 6, no. 1, pp. 117-122, 2017.

[12] S. Cheddie, C. G. Manneh, B. M. Owczarek, and Y. Moodley, "Age is a predictor of significant endoscopic findings in dyspepsia patients in South Africa," *South African Journal of Surgery,* Article vol. 58, no. 1, pp. 14-17, 2020, doi: 10.17159/2078-5151/2020/V58N1A2814.

[13] R. Sun *et al.*, "The participation of basolateral amygdala in the efficacy of acupuncture with deqi treating for functional dyspepsia," *Brain Imaging and Behavior,* vol. 15, no. 1, pp. 216-230, 2021.

[14] J. J. Peralta-Palmezano and R. Guerrero-Lozano, "Prevalence of functional gastrointestinal disorders in school children and adolescents," *The Korean Journal of Gastroenterology,* vol. 73, no. 4, pp. 207-212, 2019.

[15] M. Janota, V. Kovess-Masfety, C. Gobin-Bourdet, and M. M. Husky, "Use of mental health services and perceived barriers to access services among college students with suicidal ideation," *Journal of Behavioral and Cognitive Therapy,* Article vol. 32, no. 3, pp. 183-196, 2022, doi: 10.1016/j.jbct.2022.02.003.

[16] D. R. Patel, C. Feucht, K. Brown, and J. Ramsay, "Pharmacological treatment of anxiety disorders in children and adolescents: A review for practitioners," *Translational Pediatrics,* Review vol. 7, no. 1, pp. 23-35, 2018, doi: 10.21037/tp.2017.08.05.

[17] T. Oshima and H. Miwa, "Epidemiology of functional gastrointestinal disorders in Japan and in the world," *Journal of neurogastroenterology and motility,* vol. 21, no. 3, p. 320, 2015.

[18] A. D. Sperber *et al.*, "Worldwide prevalence and burden of functional gastrointestinal disorders, results of Rome Foundation Global Study," *Gastroenterology,* vol. 160, no. 1, pp. 99-114. e3, 2021.

[19] S. J. Tshabalala, A. Tomita, and S. Ramlall, "Depression, anxiety and stress symptoms in patients presenting with dyspepsia at a regional hospital in KwaZulu-Natal province," *South African Journal of Psychiatry,* vol. 25, no. 1, pp. 1-7, 2019.

[20] F. Qeadan, J. Egbert, and K. English, "Associations between problematic internet use and substance misuse among US college students," *Computers in Human Behavior,* Article vol. 134, 2022, Art no. 107327, doi: 10.1016/j.chb.2022.107327.

[21] A. Salsabila, "Kecemasan dan Kejadian Dispepsia Fungsional," *Indonesian Journal of Nursing and Health Sciences,* vol. 2, no. 2, pp. 57-64, 2021.

[22] E. P. Muhammad, A. W. Murni, D. Sulastri, and S. Miro, "Hubungan Derajat Keasaman Cairan Lambung dengan Derajat Dispepsia pada Pasien Dispepsia Fungsional," *Jurnal Kesehatan Andalas,* vol. 5, no. 2, 2016.

[23] P. Gajdos, N. Román, I. Tóth-Király, and A. Rigó, "Functional gastrointestinal symptoms and increased risk for orthorexia nervosa," *Eating Weight Disord.,* Article vol. 27, no. 3, pp. 1113-1121, 2022, doi: 10.1007/s40519-021-01242-0.

[24] X. Luo *et al.*, "Chinese Herbal Medicine for Functional Dyspepsia With Psychological Disorders: A Systematic Review and Meta-Analysis," *Frontiers in Neuroscience,* Review vol. 16, 2022, Art no. 933290, doi: 10.3389/fnins.2022.933290.

[25] K. Garcia-Etxebarria *et al.*, "A survey of functional dyspepsia in 361,360 individuals: Phenotypic and genetic cross-disease analyses," *Neurogastroenterology and Motility,* Article vol. 34, no. 6, 2022, Art no. e14236, doi: 10.1111/nmo.14236.

[26] A. Shah, N. J. Talley, and G. Holtmann, "Current and Future Approaches for Diagnosing Small Intestinal Dysbiosis in Patients With Symptoms of Functional Dyspepsia," *Frontiers in Neuroscience,* Review vol. 16, 2022, Art no. 830356, doi: 10.3389/fnins.2022.830356.

[27] L. Sweeney, R. Moss-Morris, W. Czuber-Dochan, and C. Norton, "Pain management in inflammatory bowel disease: feasibility of an online therapist-supported CBT-based self-management intervention," *Pilot and Feasibility Studies,* Article vol. 7, no. 1, 2021, Art no. 95, doi: 10.1186/s40814-021-00829-9.

[28] M. A. L. van Tilburg, D. A. Drossman, and S. R. Knowles, "Psychogastroenterology: The brain-gut axis and its psychological applications," *J. Psychosom. Res.,* Editorial vol. 152, 2022, Art no. 110684, doi: 10.1016/j.jpsychores.2021.110684.

[29] L. S. Walker *et al.*, "Internet-delivered cognitive behavioral therapy for youth with functional abdominal pain: a randomized clinical trial testing differential efficacy by patient subgroup," *Pain,* Article vol. 162, no. 12, pp. 2945-2955, 2021, doi: 10.1097/j.pain.0000000000002288.

[30] J. E. Wildes, A. Bedell, A. K. Graham, and M. Kells, "Brain-gut psychotherapies: Promising tools to address gastrointestinal problems in patients with eating disorders," *International Journal of Eating Disorders,* Article vol. 54, no. 6, pp. 1063-1067, 2021, doi: 10.1002/eat.23555.

[31] E. Hernandez-Ruiz, "Music to decrease anxiety in college students during the COVID-19 pandemic," *Arts in Psychotherapy,* Article vol. 80, 2022, Art no. 101953, doi: 10.1016/j.aip.2022.101953.

[32] S. Kadian, J. Joseph, S. Pal, and R. Devi, "Brief resilience interventions for mental health among college students: Randomized controlled trial," *Asian Journal of Social Health and Behavior,* Article vol. 5, no. 3, pp. 131-137, 2022, doi: 10.4103/shb.shb-28-22.

[33] A. D. Marak and B. Mathew, "Traditional oral healthcare practices followed by the Garo tribe of West Garo Hills District, Meghalaya," *Indian Journal of Traditional Knowledge,* Article vol. 21, no. 2, pp. 287-291, 2022. [Online]. Available: https://www.scopus.com/inward/record.uri?eid=2-s2.0-85130565596&partnerID=40&md5=d42857ed00aee7c830422c81ab61513f.

[34] T. Neangthaisong, S. Rattanakiat, W. Phimarn, S. Joeprakhon, K. Saramunee, and B. Sungthong, "Local wisdom and medicinal plant utilization of certified folk healers for therapeutic purposes in Buriram Province, Thailand," *Tropical Journal of Natural Product Research,* Article vol. 5, no. 4, pp. 678-685, 2021, doi: 10.26538/tjnpr/v5i4.15.

[35] Y. Shimizu *et al.*, "Exosomes from dental pulp cells attenuate bone loss in mouse experimental periodontitis," *Journal of Periodontal Research,* Article vol. 57, no. 1, pp. 162-172, 2022, doi: 10.1111/jre.12949.

[36] A. Nichols Hess and K. Greer, "Designing for engagement: Using the ADDIE model to integrate high-impact practices into an online information literacy course," *Communications in Information Literacy,* vol. 10, no. 2, pp. 6-6, 2016.

[37] M. Molenda, "The ADDIE Model," pp. 1-5, 2003.

KEMENTERIAN PENDIDIKAN, KEBUDAYAAN,
RISET, DAN TEKNOLOGI
UNIVERSITAS NEGERI PADANG
Jln. Prof. Dr. Hamka Kampus UNP Air Tawar Padang (25131)
Telp. 7058692 Fax. 7055628
E-mail: info@unp.ac.id Website: http://www.unp.ac.id

SURAT PERJANJIAN PELAKSANAAN
Riset Kolaborasi International -World Class University
TAHUN ANGGARAN 2022
No :4938/UN35.13/KP/2022

Pada hari ini **Kamis** tanggal **Satu** bulan **Desember** tahun **Dua ribu dua puluh dua**, kami yang bertandatangan di bawah ini :

1. **Upita Yeniza, S.Pd, M.Pd** : **Pejabat Pembuat Komitmen World Class University** yang berkedudukan di Padang, berdasarkan Keputusan Rektor Universitas Negeri Padang Nomor : 1342/KU/UN35/2022 tanggal 10 Juni 2022 untuk selanjutnya disebut **PIHAK PERTAMA**;

Menugaskan kepada :
2. **Dr. Zadrian Ardi, S.Pd, M.Pd, Kons** :Selaku Ketua Peneliti, beralamat di Jurusan Bimbingan Konseling FIP dalam hal ini bertindak untuk dan atas nama peneliti/kelompok peneliti, selanjutnya disebut **PIHAK KEDUA**.

Perjanjian pelaksanaan penelitian ini berdasarkan kepada :
1. Undang-Undang Republik Indonesia Nomor 17 Tahun 2003, tentang Keuangan Negara.
2. Undang-Undang Republik Indonesia Nomor 20 Tahun 2003, tentang Sistem Pendidikan Nasional.
3. Undang-Undang Republik Indonesia Nomor 01 Tahun 2004, tentang Perbendaharaan Negara.
4. Undang-Undang Republik Indonesia No. 15 Tahun 2004, tentang Pemeriksaan Pengelolaan dan TanggungJawab Keuangan Negara.
5. Undang-Undang Republik Indonesia Nomor 12 Tahun 2012 tentang Pendidikan Tinggi.
6. Peraturan Presiden Nomor 47 Tahun 2009, tentang Pembentukan dan Organisasi Kementerian Negara sebagaimana telah dirubah terakhir dengan Peraturan Presiden Nomor 77 Tahun 2011.
7. Peraturan Pemerintah No. 114 Tahun 2021 tentang Perguruan Tinggi Negeri Badan Hukum Universitas Negeri Padang
8. Permenristek dikti Nomor 44 tahun 2015; tentang Standar Nasional Pendidikan Tinggi.
9. Peraturan Menpan RB Nomor : 17 Tahun 2013 tentang jabatan Fungsional Dosen dan Angka Kredit.
10. Peraturan Pemerintah Nomor 4 tahun 2014 tentang Penyelenggaraan Pendidikan Tinggi dan Pengelolalan Perguruan Tinggi..
11. Panduan pelaksanaan Penelitian Universitas Negeri Padang tahun 2022.
12. Kontrak antara Direktur Kelembagaan Direktorat Jenderal Pendidikan Tinggi Riset, Dan Teknologi Kementerian Pendidikan, Kebudayaan, Riset, Dan Teknologi dengan Universitas Negeri Padang tentang Program Dana Abadi Perguruan Tinggi Nomor 2833/E3/KB.07.02/2022, 2056/UN.35/TU/2022 Tanggal 7 Juni 2022.
13. Peraturan Rektor Universitas Negeri Padang Nomor 10 Tahun 2022 tentang Pengelolaan Penelitian Universitas Negeri Padang.

PIHAK PERTAMA dan **PIHAK KEDUA** secara bersama-sama bersepakat mengikatkan diri dalam suatu Perjanjian Pelaksanaan Kegiatan Penelitian Program Dana Perguruan Tinggi WCU UNP Tahun Anggaran 2022 , jangka waktu pelaksanaannya dimulai setelah kontrak ditandatangani, sampai **31 Mei 2023**, dengan ketentuan dan syarat-syarat yang diatur dalam pasal-pasal berikut :

PASAL 1

(1) **PIHAK PERTAMA** memberi tugas kepada **PIHAK KEDUA**, dan **PIHAK KEDUA** menerima tugas tersebut sebagai penanggungjawab pelaksana Kegiatan Penelitian dengan judul "PENGEMBANGAN MODEL INTERVENSI KONSELING PADA REMAJA WANITA DENGAN KONDISI FUNCTIONAL GASTROINTESTINAL DISORDERS (FGID) SERTA GENERALIZED ANXIENTY DISORDER (GAD) BERBASIS PENDEKATAN COGNITIVE BEHAVIOR THERAPY, KONSELING ONLINE, DAN KEARIFAN LOKAL: STUDI CROSS-CULTURAL PADA MAHASISWA INDONESIA DAN AFRIKA SELATAN" sebagaimana tersebut di atas.

(2) **PIHAK KEDUA** bertanggung jawab penuh atas pelaksanaan, administrasi dan keuangan atas pekerjaan sebagai dimaksud pada ayat (1) dan berkewajiban menyimpan semua bukti-bukti pengeluaran serta dokumen pelaksanaan lainnya.

(3) Pelaksanaan Kegiatan Penelitian sebagaimana dimaksud pada ayat (1) dibebankan pada Program Dana Abadi Perguruan Tinggi WCU UNP sesuai keputusan Direktur Kelembagaan Direktorat Jenderal Pendidikan Tinggi Riset, Dan Teknologi Kementerian Pendidikan, Kebudayaan, Riset, Dan Teknologi dengan Universitas Negeri Padang tentang Program Dana Abadi Perguruan Tinggi Nomor : 2833/E3/KB.07.02/2022, Nomor 2056/UN.35/TU/2022 Tanggal 7 Juni 2022.

(4) Nama dosen/peneliti, judul dan besarnya biaya penelitian telah disetujui untuk didanai sesuai dengan Surat Keputusan Rektor Universitas Negeri Padang Nomor : 1212/UN35.13/KP/2022 tanggal 28 November 2022.

(5) Jangka waktu pelaksanaan penelitian oleh **PIHAK KEDUA** sudah dapat dimulai semenjak keluarnya surat keputusan dari Rektor Universitas Negeri Padang sampai dengan tanggal 31 Mei 2023, dengan rincian sebagai berikut :
   a. Penyerahan laporan kemajuan pelaksanaan penelitian dan laporan penggunaan keuangan (70%) 1 (satu) rangkap.
   b. Mengunggah laporan keuangan 70% ke **http://sim.lp2m.unp.ac.id** tanggal **6 s.d 10 April 2023.**
   c. Penyerahan laporan akhir kegiatan dan penggunaan keuangan (100%) 1 (satu) rangkap.
   d. Mengunggah laporan akhir, laporan keuangan 100%, Surat Pertanggungjawaban Belanja (SPTB). dan luaran wajib maupun tambahan ke **http://sim.lp2m.unp.ac.id** tanggal **22 s.d 31 Mei 2023.**
   e. Mengisi *logbook* penelitian pada **http://sim.lp2m.unp.ac.id.**

PASAL 2

(1) **PIHAK PERTAMA** memberikan bantuan dana untuk kegiatan sebagaimana dimaksud dalam pasal 1 sebesar ***Rp.100.000.000,- (Seratus juta rupiah)*** sudah termasuk pajak-pajak sesuai peraturan yang berlaku, yang dibebankan kepada Program Dana Abadi Perguruan Tinggi WCU UNP Nomor : 2833/E3/KB.07.02/2022, Nomor 2056/UN.35/TU/2022 Tanggal 7 Juni 2022.

(2) Dana Pelaksanaan Kegiatan **Riset Kolaborasi International - World Class University** sebagaimana dimaksud pada ayat (1) dibayarkan oleh **PIHAK PERTAMA** kepada **PIHAK KEDUA** secara bertahap melalui Bendahara Pengeluaran Pembantu UNP ke rekening Ketua Peneliti (LS).

(3) **PIHAK KEDUA** wajib menyimpan Laporan Kemajuan Pelaksanaan Kegiatan, Laporan Penggunaan Keuangan, Berita Acara Serah Terima Laporan Pelaksanaan Kegiatan, dan dokumen-dokumen Kegiatan Penelitian Hibah Riset Kolaborasi Internasional *World Class University* Tahun Anggaran 2022.

(4) **PIHAK KEDUA** bertanggungjawab mutlak dalam pembelanjaan dana tersebut pada pasal 1 ayat (1) sesuai dengan proposal kegiatan yang telah disetujui dan berkewajiban untuk

menyimpan semua bukti-bukti pengeluaran sesuai dengan jumlah dana yang diberikan oleh **PIHAK PERTAMA**.

(5) **PIHAK KEDUA** berkewajiban mengembalikan sisa dana yang tidak dibelanjakan ke Kas Universitas Negeri Padang.

(6) **PIHAK KEDUA** berkewajiban menyampaikan foto copy bukti pengembalian Dana ke Universitas Negeri Padang kepada **PIHAK PERTAMA.**

PASAL 3

(1) Dana kegiatan Penelitian ***«Skema»*** sebagaimana dimaksud Pasal 2 ayat (1) dibayarkan melalui sistem *payroll* ke rekening Ketua Peneliti oleh Bendahara Pengeluaran Pembantu UNP.

(2) **PIHAK PERTAMA** tidak bertanggungjawab atas keterlambatan dan/atau tidak terbayarnya sejumlah dana sebagaimana dimaksud dalam pasal 2 ayat (1) yang disebabkan karena kesalahan/kelalaian **PIHAK KEDUA** dalam menyampaikan data dan persyaratan lainnya yang diperlukan untuk proses pencairan dana.

PASAL 4

(1) **PIHAK KEDUA** berkewajiban memenuhi luaran wajib dan dianjurkan memenuhi luaran tambahan yang telah ditetapkan pada kontrak ini.

(2) Luaran wajib yang harus dipenuhi dalam skema penelitian sebagaimana dimaksud pada pasal 1 ayat 1 adalah :

1. ***"Publikasi minimal 1 (satu) artikel ilmiah dalam jurnal internasional bereputasi terindeks Scopus dengan kuartil 3 (Q3, SJR) dan prosiding international terindeks Scopus dengan ketua peneliti sebagai penulis pertama" .***
2. ***"Melampirkan bukti mitra pebnelitian dari perguruan tinggi luar negeri QS 400/400 by subject berkontribusi dalam survey QS academic reputasi Th 2023 dengan mengisi survey dan memilih UNP".***

(3) **PIHAK KEDUA** berkewajiban menuliskan ucapan terima kasih pada luaran Penelitian dengan mencantumkan nama Program Dana Abadi Perguruan Tinggi WCU UNP dan nomor kontrak penelitian. Contoh ucapan terima kasihnya adalah :

ACKNOWLEDGMENT

The authors would like to thank Program Dana Abadi Perguruan Tinggi WCU UNP for funding this work with a contract number : 2833/E3/KB.07.02/2022, 2056/UN.35/TU/2022.

UCAPAN TERIMA KASIH

Peneliti menyampaikan penghargaan yang tinggi dan terima kasih kepada Program Dana Abadi Perguruan Tinggi WCU UNP yang telah membiayai penelitian ini dengan nomor kontrak penelitian : Nomor 2833/E3/KB.07.02/2022, Nomor 2056/UN.35/TU/2022.

(4) Pihak Kedua Berkewajiban mengalihkan pemegang hak cipta kepada Universitas Negeri Padang yang merupakan luaran penelitian yang didanai oleh Universitas Negeri Padang.

PASAL 5

(1) Apabila **PIHAK KEDUA** berhenti dari jabatannya, sebelum pelaksanaan perjanjian ini selesai, maka **PIHAK KEDUA** wajib menyerahterimakan tanggung jawabnya kepada salah satu anggota peneliti untuk menggantikannya.

(2) Apabila **PIHAK KEDUA** tidak dapat melaksanakan tugas sebagaimana dimaksud dalam pasal 1 maka harus mengembalikan dana yang telah diterimanya ke Bendahara Pengeluaran UNP serta menyerahkan fotocopy bukti pengembalian kepada **PIHAK PERTAMA.**

(3) Apabila **3 (tiga) hari** setelah batas waktu penyampaian laporan kemajuan 70 % ini **PIHAK KEDUA** belum menyerahkan laporan hasil penelitian 70% dan laporan keuangan, maka

PIHAK KEDUA dikenakan denda sebesar **5%** (**lima persen**) dari Surat Perjanjian Pelaksanaan Penelitian tersebut,

(4) Apabila **3 (tiga) hari** setelah batas waktu penyampaian laporan akhir ini **PIHAK KEDUA** belum menyerahkan laporan hasil penelitian dan luaran wajib , maka **PIHAK KEDUA** dikenakan denda sebesar **5%** (**lima persen**) dari Surat Perjanjian Pelaksanaan Penelitian tersebut.

(5) Apabila **sampai tanggal 3 Juni 2023, PIHAK KEDUA** belum menyerahkan laporan hasil penelitian dan luaran wajib , :maka

  a) PIHAK PERTAMA tidak akan menyalurkan dana Penelitian Tahap II sebesar **30% (tiga puluh persen).**

  b) PIHAK KEDUA tidak diperbolehkan mengikuti program penelitian yang didanai WCU UNP selanjutnya.

PASAL 6

PIHAK KEDUA berkewajiban memungut dan menyetor pajak ke Rekening Kas Negara yang berkenaan dengan kewajiban pajak berupa :

(1) Pembelian barang ≥ Rp. 2.000.000,- dikenakan PPN sebesar 11% dan PPh pasal 22 sebesar 1,5% atau 3%, dengan ketentuan meminta faktur pajak dari penyedia jasa.Jika Penyedi a Jasa tidak bisa memberikan faktur pajak maka tidak di kenakan PPN.

(2) Belanja jasa dikenakan PPh pasal 23 sebesar 2% atau 4% mulai dari Rp. 0,-.

(3) Belanja jasa ≥ Rp. 1.000.000,- dikenakan PPN sebesar 10% dan dikenakan PPh pasal 23 sebesar 2% atau 4%.

(4) Belanja honorarium dikenakan PPh Pasal 21 dengan ketentuan :

  a. 5% bagi yang memiliki NPWP dan 6% bagi yang tidak memiliki NPWP untuk golongan III.

  b. 15% bagi yang memiliki NPWP dan 18% bagi yang tidak memiliki NPWP untuk golongan IV.

(5) Pajak-pajak lain sesuai ketentuan yang berlaku.

PASAL 7

(1) Apabila terjadi perselisihan antara **PIHAK PERTAMA** dan **PIHAK KEDUA** dalam pelaksanaan perjanjian ini akan dilakukan penyelesaian secara musyawarah dan mufakat dan apabila tidak tercapai penyelesaian secara musyawarah dan mufakat maka penyelesaian dilakukan melalui proses Hukum yang berlaku dengan memilih domisili Hukum di Pengadilan Negeri Padang.

(2) Hal-hal yang belum diatur dalam perjanjian ini akan diatur kemudian oleh kedua belah pihak secara musyawarah.

PASAL 8

Surat Perjanjian Pelaksanaan Kegiatan Penelitian Program Dana Abadi Perguruan Tinggi WCU UNP Tahun Anggaran 2022, ini dibuat rangkap 2 (dua) bermaterai cukup sesuai dengan ketentuan yang berlaku, dan biaya materai dibebankan kepada **PIHAK KEDUA**.

**PIHAK PERTAMA,**	**PIHAK KEDUA,**
Signed	*Signed*
**Upita Yeniza, S.Pd, M.Pd**	**Dr. Zadrian Ardi, S.Pd, M.Pd, Kons**
NIP.197501121999032001	NIDN.0001069001
